# Recurrent Primary Intrasellar Paraganglioma

**DOI:** 10.1155/2020/2580160

**Published:** 2020-06-26

**Authors:** Elizabeth A. Schueth, Daniel C. Martinez, Charles G. Kulwin, Jose M. Bonnin, Troy D. Payner, Jonathan Y. Ting

**Affiliations:** ^1^Indiana University School of Medicine, 1120 W. Michigan Street, Suite 200, Indianapolis, IN 46202, USA; ^2^Department of Otolaryngology – Head and Neck Surgery, Indiana University School of Medicine, 1130 W. Michigan Street, Fesler Hall, Suite 400, Indianapolis, IN 46202, USA; ^3^Goodman Campbell Brain and Spine, 13345 Illinois Street, Carmel, IN 46032, USA; ^4^Department of Pathology and Laboratory Medicine, Indiana University School of Medicine, 350 W. 11^th^ Street, Indiana University Health Pathology Laboratory, Indianapolis, IN 46202, USA

## Abstract

We describe a case of an 81-year-old male presenting with bitemporal visual field defects and blurry vision in the right eye. The patient was found to have a recurrent primary paraganglioma in the sellar and suprasellar region requiring a repeat transsphenoidal endoscopic resection. Immunohistochemical examination confirmed paraganglioma with the classic zellballen appearance which stained positive for chromogranin, synaptophysin, and S-100 in the periphery. Paragangliomas (PGLs) in the sella turcica are a rare entity; only 19 cases have ever been reported in the literature. PGLs in the sellar region are often misdiagnosed or diagnosed in a delayed fashion. Earlier diagnosis of this locally aggressive tumor and meticulous debulking can prevent morbidity secondary to the tumor's compressive effects. This report highlights the effectiveness of surgical interventions in treatment of paragangliomas. More research is still needed to determine the need for adjuvant therapies such as radiation.

## 1. Introduction

Paragangliomas (PGLs) are rare, nonepithelial neuroendocrine neoplasms (NENs) [1] that arise from neural crest cells and generally occur in locations where paraganglia normally are found [2]. There are two main types: parasympathetic and sympathetic [3]. Parasympathetic are usually in the head and neck and nonfunctional, while sympathetic are typically in the thorax, abdomen, retroperitoneum, and pelvis and are functional [3–5]. The most well-known type of paraganglioma is a pheochromocytoma which originates in the adrenal medulla [5]. While all PGLs have neurosecretory granules, those classified as functional secrete catecholamines leading to symptoms such as secondary hypertension, flushing, increased perspiration, headaches, and tremors [6]. Only 1–3% of PGLs in the head and neck are functional [7].

In the head and neck, PGLs account for only 0.6% of all tumors [8]. Of all PGLs, only 3% occur in the head and neck region [7]. PGLs are found along parasympathetic paraganglia of the glossopharyngeal and vagus nerves with the most common site being the carotid body (75%) and the second being the temporal bone (16%) [3, 8, 9]. Other sites such as vagal, laryngeal, subclavian, aorticopulmonary, and cardiopulmonary are also documented [3].

60–70% of carotid body PGLs present as a nontender, enlarging mass in the lateral neck often with a bruit and can lead to cranial nerve IX, XI, and XII deficits [7]. Vagal PGLs are also nontender, slow growing neck masses in the lateral neck with the majority at the angle of the mandible. Symptoms consist of pulsatile tinnitus and in one to two thirds lower cranial nerve palsies in X, IX, XI, and XII [7].

When PGLs are intracranial, over 90% are from the temporal bone, in the jugular foramen or middle ear (termed tympanic) [10]. Jugular PGLs present as an enlarging mass around the jugular bulb leading to decreased venous blood return on that side and resultant rerouting to the contralateral sigmoid sinus [7]. These often lead to pulsatile tinnitus, an audible bruit over the ear, dizziness, or hearing loss [7]. Tympanic PGLs typically present as a small mass in the tympanic membrane or external auditory canal associated with tinnitus and hearing loss [7]. PGLs generally present as an enlarging mass with symptoms based on the topographic location.

Sellar PGL generally presents as an enlarging sellar mass most often associated with vision loss/impairment and headaches [11]. To our knowledge, there are only 19 cases reported of primary sellar paragangliomas. We present a case of a recurrent paraganglioma of the sellar and suprasellar region and compare our findings to the current literature.

## 2. Case Presentation

An 81-year-old male with a history of atrial fibrillation on warfarin, hypertension, and glaucoma initially presented with progressively worsening blurry vision. On physical examination, he was found to have visual field defects, with no other cranial nerve sensory or motor defects. Additionally, there was no evidence of endocrinopathy or autonomic changes noted.

On radiologic evaluation, the patient was found to have a sellar mass pressing on the optic chiasm, most consistent with a pituitary microadenoma. Two years earlier, he underwent a transseptal transsphenoidal resection of the pituitary tumor at another institution to decompress the optic chiasm. Histopathological evaluation of the resection had features of a paraganglioma. Postoperatively, there was no progression of his visual deficits.

Several months later, the patient presented to our clinic with worsening visual problems especially blurry vision on the right side and decreased short-term memory. He also reported two episodes of epistaxis within the prior ten days. He denied having any problems with balance or episodes of headaches. On physical examination, he had decreased bitemporal fields and an increased right inferior field but can still see hand movements in both superior quadrants on the right side. Visual acuity in the left eye was good. The pupils were equal, round, and reactive to light. The extraocular movements were intact. The remainder of the physical exam was unremarkable.

**Figure 1:**
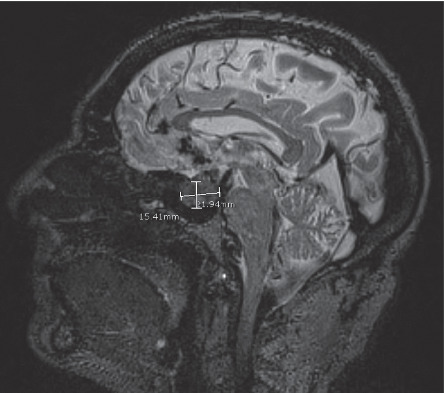
Preoperative sagittal T2-weighted MRI showing 15.41 mm by 12.94 mm paraganglioma in the sellar and suprasellar region.

On imaging, MRI demonstrated a lobulated 1.9 cm × 2.1 cm × 2.3 cm T2 hyperintense homogeneous enhancing mass causing expansion of the sella turcica and extending into the suprasellar cistern compressing the optic chiasm and the right optic nerve (Figure 1). There was also extension into the medial aspect of the cavernous sinus.

**Figure 2:**
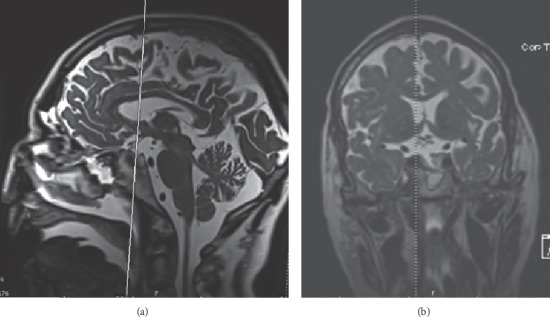
Intraoperative T2-weighted MRI: (a) sagittal and (b) coronal views following resection of paraganglioma with the cross-sectional slice indicated by the vertical lines.

Transsphenoidal endoscopic resection with intraoperative MRI and CT stealth stereotactic navigation was used to extensively decompress the optic nerve and chiasm and resect the paraganglioma. Intraoperative MRI demonstrated a gross-total resection of the tumor (Figures 2(a) and 2(b)).

**Figure 3:**
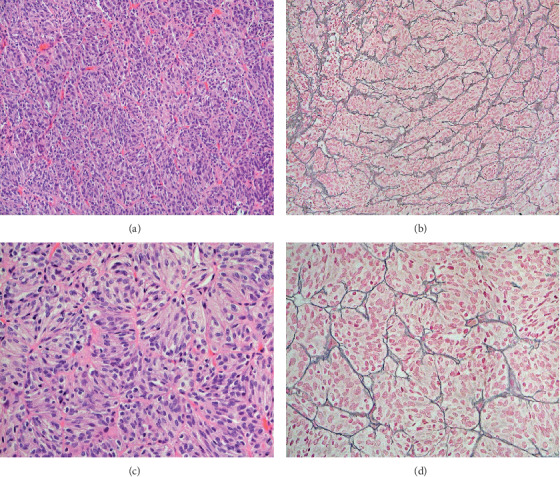
Paraganglioma with a distinct nested pattern of the tumor cells (“zellballen”) in sections stained with hematoxylin-eosin (H&E) (a, c); the nested pattern was highlighted by a silver impregnation for reticulin fibers (b, d). Figures a and b 100x; figures b and c 400x.

**Figure 4:**
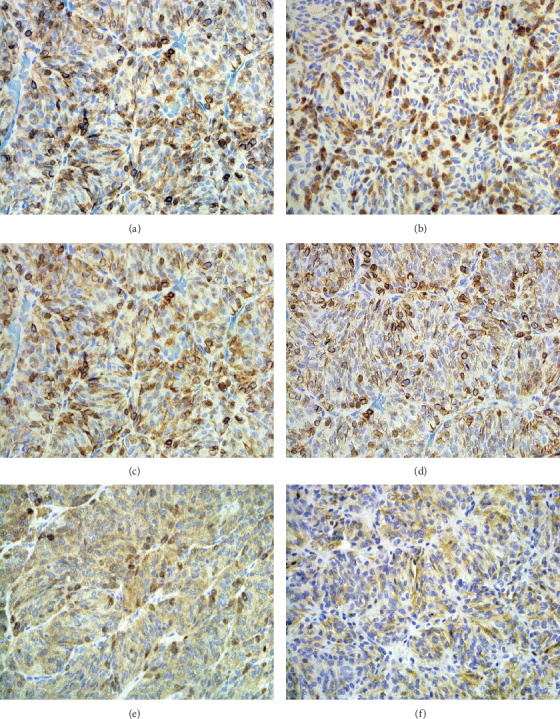
Strong immunoreactivity in principal cells for synaptophysin (a), chromogranin (c), CD56 (d), and neuron-specific enolase (e). Sustentacular cells with strong immunoreactivity for S-100 protein (b). Focally, the tumor cells were also positive for AE1/AE3 (cytokeratin) (f); figures a–f, 200x.

On histopathological evaluation, the tumor displayed features of a paraganglioma. The tumor cells were arranged in relatively well-defined nests (“zellballen”) (Figures 3(a) and 3(c)) surrounded by a delicate network of reticulin fibers. These were highlighted in the sections stained with the silver impregnation method (Figures 3(b) and 3(d)). Relatively large elongated cells, with clear cytoplasm (principal cells), were the most prominent component of nests. The sustentacular cells were difficult to detect in the routine stained sections. Only a rare mitotic figure was identified, and areas of necrosis were not observed. By immunohistochemistry, the tumor cells were positive for synaptophysin (Figure 4(a)), chromogranin (Figure 4(c)), NCAM (neural cell adhesion molecule, CD56) (Figure 4(d)), and neuron-specific enolase (Figure 4(e)). The sustentacular cells were strongly immunoreactive for S100 protein (Figure 4(b)). Focally, the tumor cells were also positive of cytokeratin (AE1/AE3) (Figure 4(f)). They were not immunoreactive for calretinin or neurofilament protein.

The patient is currently symptom-free with no recurrent tumor on repeat imaging 2.5 years after surgery.

## 3. Discussion

Paragangliomas arise from neural crest cells, but the exact pathogenesis of sellar PGLs is not completely understood. A widely accepted hypothesis indicates paraganglionic cells from cranial nerve IX (tympanic or ciliary branches) migrate into the cavernous sinus that is just adjacent to the sella turcica [12].

The differential diagnosis for sellar tumors is large and includes pituitary adenomas (85%), craniopharyngiomas (3%), Rathke cleft cysts (2%), meningiomas (1%), and metastases (0.5%) [13]. Other rare masses such as oncocytomas, neurohypophysis granular cell tumors, hypophysitis, and pituicytomas are also seen [14]. Paragangliomas in this region are an extremely rare diagnosis and often initially misdiagnosed.

According to a recent literature review, sellar paragangliomas are most commonly initially diagnosed as a pituitary adenoma (77.8%) followed by meningioma, craniopharyngioma, and macroadenoma [11]. Of the 19 other sellar PGL case reports, 9 were initially misdiagnosed (4 as adenomas, 2 as meningiomas, 2 as macroadenomas, and 1 as a craniopharyngioma while 10 other cases did not report on misdiagnosis) [11]. Our patient was initially misdiagnosed with a pituitary adenoma.

The most common presenting symptom for sellar PGLs, as seen with our patient, is visual disturbances. Of the 19 sellar PGLs, patients' symptoms on presentation were visual disturbances/vision loss (63.2%, 12/19), headaches (47.4%, 9/19), hyperhidrosis (10.5%, 2/19), and one patient (5.3%, 1/19) with each of the following: gait imbalance, amaurosis, depression, anxiety, amenorrhea, proptosis, conjunctival vessel dilation, impotence, asthenia, dysthymic disorder, temporospatial confusion, abnormal behavior, galactorrhea, confusion, short-term memory loss, progressive lower limb hyposthenia, vertical nystagmus, arrested growth and sexual development, and hypopituitarism [11].

The typical workup, if the patient is suspected to have a PGL or pheochromocytoma, consists of an MRI or CT of the abdomen and pelvis, a serum calcitonin level, 24-hour urine collection to measure catecholamine levels, and plasma-free fractionated metanephrines [6]. Of head and neck PGLs, 6–19% are malignant [15]. The diagnosis of malignant paraganglioma is currently debated. Studies have indicated that histopathological features (hypervascularity, central necrosis, and increased mitotic activity) are unreliable in distinguishing benign from malignant PGLs [2]. The currently accepted definition is when the tumor has spread to other nonendocrine tissues [2, 16].

Since only 1–3% of head and neck PGLs are functional, they generally do not result in increased catecholamine levels which cause the elevated blood pressures, flushing, palpitations, and so on [7]. As described in the introduction, most patients with head and neck PGLs initially present with symptoms related to mass effect based on topographic location or incidental finding on imaging [4]. Carotid and vagal PGLs present with enlarging masses and lower cranial nerve palsies [7]. Vagal, jugular, and tympanic PGLs can all lead to hearing loss and tinnitus [7]. And as seen in our patient, visual disturbances are the most common initial presenting symptom in sellar PGLs (65%, 13 of 20 sellar cases) [11].

The average age of presentation is the 5^th^-6^th^ decade for carotid and jugulotympanic, 5^th^ decade for vagal, and 4^th^–6^th^ decade for laryngeal PGLs [17]. Of the 20 sellar cases including this report, the average age with standard deviation was 48.3 ± 21.1 years with a wide range from 14 to 84 years. The female to male ratio is 2 : 1 (8 : 1 in high altitude) for carotid, 2:1–8:1 for vagal, 3:1–9:1 for tympanic, and 3 : 1 for laryngeal PGLs [17]. On the contrary, 20 sellar PGLs had a male predilection with 14 males (70%) and 6 females (30%; M : F ratio of 2.3 : 1). Bilateral and/or multifocal PGLs occur in 10–25% of carotid, 20–40% of vagal, often with carotid and/or vagal PGLs for tympanic, and rarely for laryngeal PGLs [17]. None of the 20 sellar PGLs, including the present study, were reported to be bilateral or multifocal [11]. The metastatic risk also varies by type: 4–6% in carotid, 16% in vagal (metastatic vs. multifocal was not clear), 2% in tympanic, and 2% in laryngeal [17]. Only 1 of the 20 sellar PGLs (5%) was noted to have metastasis [14]. Additionally, none of the 20 sellar PGLs had elevated catecholamine levels [11, 12].

CT and MRI are the most common imaging studies used in diagnosis with sensitivity of 95–100% and specificity of 67–70% [18]. PGLs enhance on contract CT and gadolinium MRI [19]. PGLs have low signal on T1-weighted and high signal on T2-weighted MRI, as seen in our patient's hyperintense homogeneous sellar PGL (Figure 1) [19]. One classic sign, seen more in head and neck PGLs than those in the trunk, is a “salt and pepper” appearance composed of flow voids creating low-signal intensity and hemorrhage creating hyperintense regions on both T1- and T2-wieghted MRI [20]. This “salt and pepper” pattern is not seen in any of the 20 sellar PGLs, but rather sellar PGLs are described as a well-circumscribed homogeneous or heterogeneous mass with intense contrast enhancement [12]. Only 2 of the 20 sellar cases described flow voids [14, 21]. Most head and neck patients have nonfunctional PGLs; thus, biomarker imaging that is commonly used for functional PGLs of the abdomen is not always useful [19]. However, if the patient's head and neck PGL makes catecholamines, then 3-methoxytyramine may be used to localize these tumors [19]. Our patient had a nonfunctional PGL, so biomarker imaging was not utilized.

10–50% of paragangliomas are secondary to a hereditary condition such as familial paraganglioma, neurofibromatosis type I, von Hippel–Lindau disease, Carney triad, and occasionally, multiple endocrine neoplasia type 2 (MEN2) [4]. The most common genetic mutation with benign paragangliomas of the head and neck is the succinate dehydrogenase (SDH) mitochondrial complex [17]. The mutational status for SDH was not investigated in this case. For head and neck PGLs, patients with a genetic mutation most commonly present with a family history, multiple lesions/malignant, and are 40 years or younger [7].

Diagnosis is made based on histopathology with the classic description of “zellballen” or nesting of cells surrounded by abundant capillaries which are found in normal paraganglia [22]. In the periphery, sustentacular cells stain positive for S-100 protein [22] which was seen in our patient and is reported to occur in over 50% of the cases in the literature [11]. In the prior literature, all sellar paragangliomas have been chromogranin- and synaptophysin-positive as in our patient.

After diagnosis, management of head and neck PGLs differs based on the type. For functional PGLs or pheochromocytomas, patients will require preoperative blood pressure managements with alpha-blockers (typically phenoxybenzamine) and beta-blockers (propranolol) to avoid intraoperative hypertensive crisis [18]. Primary treatment is tumor resection which is widely agreed upon. Carotid PGLs with SDHB mutations are more likely to develop local/regional recurrence and/or distant metastases and may require more extensive surgery [23]. Adjuvant therapy with radiation is currently under debate as to improved patient outcomes. Of the 20 sellar PGLs, 35% (7/20) received radiation therapy and 25% (5/20) had an additional resection: pterional (10%, 2/20), subfrontal (10%, 2/20), and frontal (5%, 1/20) [11]. One study found postoperative radiation slowed progression of residual disease in patients with malignant PGLs [16]. Additionally, patients with metastatic PGLs may benefit from chemotherapy to decrease symptoms or for tumor shrinkage [24]. The only sellar PGL patient with metastasis, to the skull and the right femur, underwent surgery and radiation but not chemotherapy [11].

There are limited data on recurrence and overall survival rates of these patients. Of the 20 sellar patients, all patients are living at time of follow-up (months to years); however, many patients have postoperative morbidity with visual disturbances in 15% (3/20), diabetes insipidus in 15% (3/20), adrenocortical insufficiency in 5% (1/20), diplopia in 5% (1/20), persistent hyposthenia in 5% (1/20), basal ganglion infarction in 5% (1/20), headaches in 5% (1/20), and hypopituitarism in 5% (1/20) [11]. Patients with single-site PGLs after surgical resection have an equivalent life expectancy to age-matched disease-free individuals [6]. However, around 30% of patients have recurrence of their paraganglioma [18]. Patients with familial forms are 3.4 times more likely to have recurrence than those with sporadic disease [25]. Of these patients with recurrence, 50% have had distant metastasis [25]. Follow-up is highly recommended since many patients have persistence or recurrence of disease. Patients should be encouraged to undergo genetic testing to identify if there is a syndromic link. Postoperatively, patients should undergo annual surveillance with a MRI or CT to ensure the tumor has not recurred [6]. Patients with functional PGLs should also undergo annual biochemical screening [6].

## 4. Conclusions

While, rare, paragangliomas should be kept on the differential for a sellar lesion. If there is a high index of suspicion based on symptoms, family history, elevated catecholamines, etc., an additional workup should be considered. Surgery is the mainstay of treatment, with no clear consensus for adjuvant therapy in the literature.
